# Use of a counsellor supported disclosure model to improve the uptake of couple HIV testing and counselling in Kenya: a quasi-experimental study

**DOI:** 10.1186/s12889-018-5495-5

**Published:** 2018-05-18

**Authors:** Margaret Kababu, Eric Sakwa, Robinson Karuga, Annrita Ikahu, Inviolata Njeri, Jordan Kyongo, Catherine Khamali, Wanjiru Mukoma

**Affiliations:** 1 LVCT Health, Nairobi, Kenya; 2 Root capital, Nairobi, Kenya; 3 Trocaire, Nairobi, Kenya

**Keywords:** Counsellor supported disclosure (CSD), Couple HIV testing and counselling (CHTC), Intervention, HIV status disclosure, HIV discordant couples

## Abstract

**Background:**

Heterosexual couples account for 44% of new HIV infections in Kenya and there’s low awareness of self and partner HIV status. Different strategies have been employed to promote couple HIV testing and counselling (CHTC). Despite this, HIV incidence among couples continues to rise. This study sought to assess the use of a counsellor-supported disclosure (CSD) model in enhancing the uptake of CHTC and the factors that were associated with it.

**Methods:**

A pre-post quasi experimental study design with an intervention and a comparison arm was utilized. The study was conducted in Nairobi, Nakuru, Kisumu, and Homa Bay counties in Kenya. A total of 276 participants were recruited; 149 and 127 in the comparison and intervention arms, respectively. Standard HIV testing & counselling (HTC) was offered in the comparison arm whereas the counsellor-supported disclosure model was administered in the intervention arm. The model empowered index clients to invite their sexual partner for CHTC. Telephone follow-up and subsequent community health volunteer (CHV) follow-up for non-responders were embedded in the model. Semi-structured questionnaires were used to collect data at baseline and 3 months into the study. In-depth interviews were conducted with 15 participants who took up the intervention and 7 of the HTC providers who offered CSD. The quantitative and qualitative data were analyzed using STATA version 13 and NVIVO 10, respectively.

**Results:**

Uptake of CHTC was 28% in the intervention arm of the study compared to 7% in the comparison arm (*p* < 0.001). Participants in the intervention arm of the study had eight times higher odds of taking up CHTC compared to their counterparts. The outcome of the qualitative interviews revealed that the CSD counselling, skills on partner invitation, and follow-up for partner invitation increased the uptake of CHTC. On the other hand, unwillingness to test together with partner, lack of availability to test together as a couple, and provision of the wrong contact information by the participants reduced the uptake of CHTC.

**Conclusion:**

The CSD model improved the uptake of CHTC. This model can be integrated into the existing HTC structures to enhance the uptake of CHTC.

## Background

Sub-Saharan Africa has the highest prevalence and incidence of heterosexually acquired HIV infection worldwide [1]. In East Africa, a high proportion of incident HIV infections have been shown to occur among married/cohabitating heterosexual couples with approximately one out of every two HIV-infected couples in a stable discordant partnership [2]. In Kenya, married couples and those in stable sexual relationships account for the highest percentage (44%) of new HIV infections [3, 4]. Knowledge of self and partner’s HIV status has also been reported to be low among heterosexual couples. According to the 2012 Kenya AIDS Indicator Survey (KAIS), 53% of persons found to be HIV-infected did not know that they were infected; and 40% of married/cohabiting couples were not aware of their partner’s HIV status [5].

HIV testing and counselling (HTC) facilitates the knowledge of HIV status and linkage to prevention, care and treatment services [6]. Couple HIV testing and counselling (CHTC) occurs when two persons who are planning to be in a sexual relationship or are in an ongoing sexual relationship are counselled, tested and receive their HIV results together [7]. CHTC provides an avenue for mutual disclosure of HIV status in an environment where support can be provided by a counsellor or health worker; risk-reduction messages can be tailored to the outcome of the test results of both individuals and decisions about prevention, accessing treatment, care and support, and family planning options can be made together decreased stigma; and normalization of HIV. Other benefits of CHTC include potential for behavior change to reduce transmission among couples; uptake and adherence to antiretroviral therapy (ART) for individuals who test positive and pre-exposure prophylaxis (PrEP) for HIV negative individuals; increased uptake and adherence to prevention of mother-to-child transmission (PMTCT) leading to decreased number of infants with HIV; increased marital cohesion and reduction of intimate partner violence (IPV); decreased stigma; and normalization of HIV [7–9]. Despite the evidence of benefits of CHTC, the uptake of couple HIV testing and counselling in Kenya remains relatively low (31.5%) [5].

Factors associated with high uptake of CHTC include prior discussions on HTC with partner, awareness of CHTC benefits, availability of time to test as a couple, positive attitude of service providers, and short distance to testing facility, partner invitation for CHTC uptake and follow-up/tracing of partners for uptake of CHTC [10–12]. Factors associated with low uptake of CHTC include conflicting work schedules, fear of negative outcomes of disclosure, unwillingness of partner to test, low risk perception of HIV infection, prior testing of HIV, marital status, and lack of CHTC awareness [11, 12, 14–16].

Different strategies have been employed to promote the uptake of CHTC. In a study conducted among women attending a Nairobi antenatal clinic to determine effect of partner involvement and couple counselling on uptake of interventions to prevent HIV-1 transmission, only 6% of the participants accepted couple HIV testing and counselling [17]. In a similar study in Zambia, only 10% of women were able to encourage their partners to take up CHTC despite community outreach activities promoting CHTC [18] while another using influence network leaders and agents yielded a 6% success rate [19]. A study by Allen et al. on promotion of couples’ voluntary counselling and testing for HIV through influential networks in two African capital cities yielded a success rate of 14.3% [20]. An unblinded randomized controlled trial conducted in Malawi to assess the uptake of CHTC in the antenatal unit at Bwaila District Hospital revealed that an invitation plus tracing strategy increased the uptake of CHTC by 22% [13].

HIV incidence among couples in Sub-Saharan Africa has continued to rise, calling for more strategies to increase the uptake of CHTC [2, 5, 21, 22]. In 2012, the World Health Organization (WHO) recommended counsellor-supported disclosure (CSD) as a key strategy that may be implemented to enhance mutual knowledge of HIV status among sexual partners and couples through a disclosure process [7]. Support for mutual disclosure has been recommended for index persons who test for HIV regardless of the HIV test outcome in high HIV prevalence settings like Sub-Saharan Africa [7]. According to the KAIS (2012), CSD is a key strategy for reducing the high rate of HIV transmission among couples [5]. Counselling techniques to facilitate disclosure of HIV status in discordant relationships have also been reported to be vital in HIV prevention among couples [23]. Supported disclosure also has the potential to mitigate the negative outcomes of individual HIV status disclosure [24].

In 2014, LVCT health developed a CSD model, dubbed the *Tunajijua* model to improve CHTC. *Tunajijua* is a Swahili word meaning ‘we know ourselves’. This study sought to establish the efficacy of the *Tunajijua* model in improving the uptake of CHTC in HIV testing and counselling sites within community and clinical settings. We also report on the factors that were associated with uptake of CHTC following the CSD intervention from both the clients and HTC service providers’ perspective.

## Methods

### Study design

**Fig. 1:**
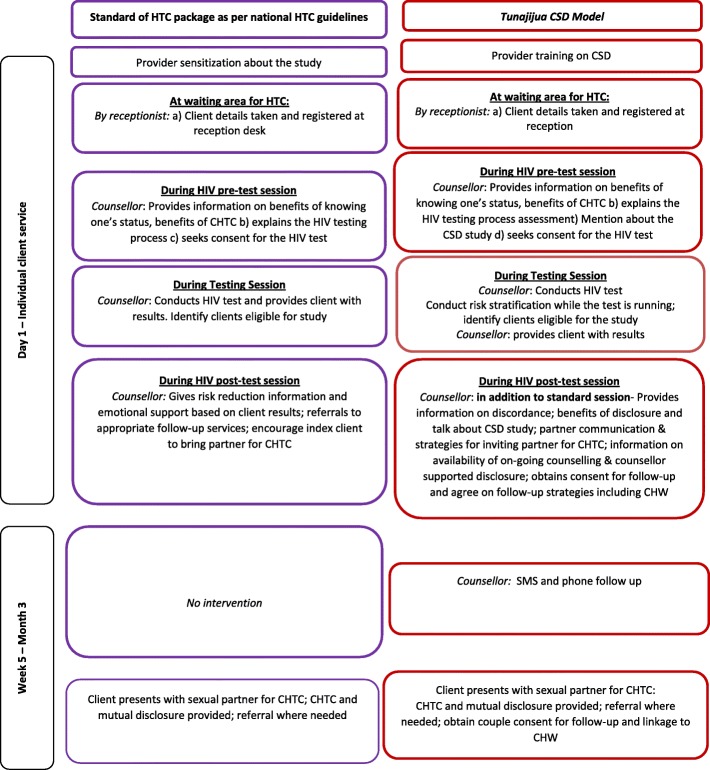
Diagrammatic representation of Standard HTC and the ‘Tunajijua’ (CSD) Model. Study participants in the comparison arm received standard HTC as described on the left panel of the diagram while those in the intervention arm received services described on the right panel of the figure i.e. CSD intervention

A pre-post quasi experimental study design with an intervention and a comparison arm was utilized in this study. The participants in the intervention arm received the standard HTC and were also taken through the CSD model whereas participants recruited in the comparison arm only received standard HTC. Details of services and procedures in each study arm are provided in Fig. 1.

### Study location

The study was conducted in LVCT Health sites and LVCT Health-supported HIV testing and counselling sites in Nairobi, Nakuru, Kisumu and Homa Bay counties. These are among the nine counties that contribute to the highest rate of new HIV infections in Kenya [25]. The sites were purposively assigned to the intervention and comparison arms of the study prior to the study inception. Participants in the intervention sites were enrolled in the study intervention whereas those in the comparison site were assigned to the comparison arm.

### Sample size, sampling procedures and eligibility criteria

We determined that a sample size of 256 clients (128 clients in each arm) would be sufficient to demonstrate a hypothesized 20% point increase in couple HIV testing after the *Tunajijua* intervention with 95% level of confidence (alpha = 0.05) and 90% power. Sample size in the comparison arm was inflated by 15% to factor in loss to follow up since the comparison arm did not involve rigorous follow up. The study ended up recruiting 127 participants in the intervention arm and 149 participants in the comparison arm.

The study targeted clients presenting alone for HTC as well as HTC counsellors involved in the implementation of the *Tunajijua* intervention. The eligibility criteria for HTC clients recruited in the study included: a) clients who tested HIV positive b) clients who were found to be at high risk of HIV infection (a person was considered to be at high risk of HIV infection if they answered ‘yes’ for any of the following: had unprotected sex with persons of unknown HIV status, had multiple sexual partners; was a man who has sex with men (MSM)/female sex worker (FSW), people who inject drugs (PWID)/truck driver; had unprotected sex with MSM/FSW/PWID/truck driver; did not know the HIV status of their sexual partner(s); had been diagnosed with or treated for a sexually transmitted infection (STI); and had been coerced/forced into having sex) c) those who reported to have had a steady sexual partner for at least six months prior to the study d) aged 18 years and above e) had good knowledge of English/Kiswahili and f) willingness to participate. Participants were consecutively enrolled into the study until the desired sample was attained. This was done between April and May 2014. In-depth interviews (IDIs) were conducted with 15 participants who had received the CSD intervention.

IDIs were also conducted with 7 HTC service providers who were purposively selected to participate in the study. The eligibility criteria for HTC service providers interviewed included being involved in the implementation of the CSD intervention and willingness to participate in the study.

### Description of the *Tunajijua* CSD model

The CSD model was built on the national standard HIV testing package with some additional components as described in Fig. 1. The model comprised of: 1) counselling 2) active partner invitation and follow-up 3) couple HTC and 4) effective referral and linkages. Five main topics were covered by counsellors during counselling, follow-up and linkage support: i) HIV information including discordance and concordance; ii) couple communication including effective partner communication, iii) benefits of disclosure and possible methods of disclosure, iv) assertive and negotiations skills, and v) self-awareness skills. The HTC counsellors at the intervention arm sites received a three-day training on the delivery of the *Tunajijua* intervention prior to initiation of the study. Training on the CSD model skilled the HIV testing counsellors on empowering the index HTC client (individual presenting alone for HTC) to invite their sexual partner for CHTC at the clinic and linking the couples to appropriate post-HIV test interventions.

### Study outcomes

Uptake of CHTC was defined as the number of participants who brought back their partner for mutual HIV testing and disclosure within three months after the initial HIV test.

### Data collection

Quantitative and qualitative data collection methods were utilized in the study. In the intervention arm of the study, quantitative data was collected using interviewer-administered semi-structured questionnaires. Data on sociodemographic characteristics, HIV knowledge and perceptions on disclosure of HIV status, and perceptions on follow-up, referral, and linkage were collected at baseline and at the 3 month follow-up. Additional information captured at follow-up included partner invitation for CHTC, uptake of CHTC and experience on the different components of the *Tunajijua* intervention (CSD counselling, follow up and referral). Qualitative data was collected from service providers who offered the CSD intervention and clients who took up CSD through in-depth interviews to explore the factors that were associated with the uptake of CHTC. All the interviews and questionnaires were administered by research assistants trained in the study protocol and had experience in qualitative and quantitative data collection.

In the comparison arm of the study, an interviewer-administered questionnaire was used to collect data from the study participants at baseline. Data on sociodemographic characteristics, HIV knowledge and perceptions on disclosure of HIV status, and perceptions on follow-up, referral, and linkage. The questionnaire was administered by trained research assistants. A data abstraction tool was used by the HTC counsellors providing services to capture data from participants who returned for CHTC within 3 months of the study. The information captured included partner invitation for CHTC, uptake of CHTC, uptake of referrals and linkage for other services. The HTC counsellors were sensitized on how to utilize the tool.

### Data analysis

Quantitative data was analysed using STATA version 13. Data quality was reaffirmed by conducting a series of checks such as range, consistency, completeness, logical, and correctness checks. Chi-square test was conducted to check for differences in sociodemographic characteristics of participants in the comparison and intervention arms of the study at baseline. Univariate analysis were conducted to establish the factors that were associated with the uptake of CHTC. All factors associated with CHTC uptake in the univariate were included in a multivariate logistic regression model to further assess if the intervention improved CHTC uptake. All missing data were excluded from the analysis. A *p* < 0.05 was considered statistically significant. Incomplete records were omitted from the analysis.

Qualitative data were transcribed verbatim, typed in MS Word, translated into English where necessary and imported into NVIVO V.10 (NVIVO qualitative data analysis software; QSR International Pty Ltd. Version 10, 2012). A framework analytical approach, guided by the study objectives, was developed and used to identify key emerging themes from the transcripts. All coded or double coded transcripts were linked by use of classifications and queries on each theme and sub theme generated to guide writing of narratives. Analysed data was presented in a descriptive manner and selected quotes illustrating common themes included in text.

## Results

### Description of the study population

Table 1 gives a description of the study population at baseline. The participants had a median age of 28 years. A significant difference (*p* = 0.021) was recorded between age of participants in the intervention and comparison arms of the study. About 50% of the participants in the intervention arm were in the age group 25–34 compared to 35% in the comparison arm of the study. Over half, 150 (54.3%), of the respondents were male, 141 (51.1%) were married, 202 (73.2%) were employed, and 114 (41.3%) had attained secondary education. Significant differences were recorded between marital status (*p* < 0.001) and level of education in the two arms of the study (p < 0.001). Sixty percent of the participants in the intervention arm had never been married whereas 63% in the comparison arm were married. Six percent of all the participants were HIV positive whereas 259 (94%) were HIV high-risk negative. A total of 253 (97.1%) study participants reported having had a HIV test prior to the study. Knowledge of partner’s HIV status was low at only 23.9% prior to the study. A significant difference (p < 0.001) was recorded on knowledge of partner’s HIV status between the two groups. Twenty eight percent of the respondents in the comparison arm were aware of their partners’ HIV status compared to 18% in the intervention arm. Most of the participants reported willingness to invite their steady partners for a HIV test (88%) and willingness to take a test together with their partners 92.8%. A significant difference (*p* = 0.002) was recorded in willingness to test together with partner in the two arms of the study. Participants in the comparison arm (97%) were more willing to test together with their partners compared to those in the intervention arm (87%).

**Table 1:** Sociodemographic and baseline characteristic of study participants

Outcome variable	Arm of study				Total		*P*-Value
	Intervention(*n* = 127)		Comparison (*n* = 149)				
	Count	%	Count	%	Count (*n* = 276)	%	
Age range							
17–24 years	34	26.8%	49	32.9%	83	30.1%	*0.021*
25–34 years	65	51.2%	52	34.9%	117	42.4%	
35–44 years	20	15.7%	41	27.5%	61	22.1%	
45 years and above	8	6.3%	7	4.7%	15	5.4%	
Median age (IQR^a^): 28 years (24–35)							
Sex of participant							
Female	55	43.3%	71	47.7%	126	45.7%	0.47
Male	72	56.7%	78	52.3%	150	54.3%	
Occupation of participant							
Unemployed	34	26.8%	40	26.8%	74	26.8%	0.989
Employed	93	73.2%	109	73.2%	202	73.2%	
Marital status							
Never married	76	59.8%	46	30.9%	122	44.2%	*< 0.001*
Married	47	37.0%	94	63.1%	141	51.1%	
Widowed/separated/divorced	4	3.1%	9	6.0%	13	4.7%	
Education							
Primary education and below	16	12.6%	60	40.3%	76	27.5%	*< 0.001*
Secondary education	38	29.9%	76	51.0%	114	41.3%	
Tertiary	73	57.5%	13	8.7%	86	31.2%	
Participant’s HIV status							
HIV high risk negative	122	96.1%	137	91.9%	259	93.8%	0.156
HIV positive	5	3.9%	12	8.1%	17	6.2%	
Prior testing for HIV							
No	9	7.1%	14	9.4%	23	8.3%	0.489
Yes	118	92.9%	135	90.6%	253	91.7%	
Knowledge of partner’s status							
No	89	70.1%	106	71.1%	195	70.7%	*< 0.001*
Yes	24	18.9%	42	28.2%	66	23.9%	
Not sure if partner status has changed	14	11.0%	1	0.7%	15	5.4%	
Willingness to invite partner for a HIV test							
No	16	12.6%	17	11,5%	33	12%	0.777
Yes	111	87.4%	131	88.5%	242	88%	
Willingness to test together with partner							
No	16	12.6%	4	2.7%	20	7.2%	*0.002*
Yes	111	87.4%	145	97.3%	256	92.8%	

*Notes*: Missing data were excluded from the analysis

^a^IQR: interquartile range

### Uptake of CHTC

A significant difference was recorded in the uptake of CHTC in the two arms of the study (*p* < 0.001). The uptake of CHTC was 28% in the intervention arm and 7% in the comparison arm of the study as illustrated in Table 2.

**Table 2:** Uptake of CHTC three months after the intervention

Outcome variable	Arm of study		*P* value
	Intervention (n = 127) n (%)	Control (n = 149) n (%)	
Uptake of CHTC			
Yes	36 (28.4)	11 (7.4)	< 0.001*
No	91 (71.6)	138 (92.7)	

*Notes*: Pearson Chi-square test was applied * p < 0.05

### Qualitative findings on uptake of CHTC

According to the service providers who offered the CDS intervention, many individuals who took up the intervention brought back their partners for CHTC as illustrated below:

*“… I have had many people who brought back their partners for CHTC… They said they came to this room for the CSD ...”* ***(Female, service provider)***

*“… Like last month we had so many couples taking up the services [CHTC] and I want to believe it is because of the issue of the CSD…”* ***(Male, service provider)***

However, one service provider felt the uptake of CHTC was not as high as they had anticipated given the introduction of CSD as quoted below:

*“…the turn up if I compare to the percentage that we were expecting the index clients to come back is a little bit low …”* ***(Male, service provider)***

### Factors associated with uptake of CHTC

The outcome of univariate analysis revealed a significant association between uptake of CHTC and study arm [OR = 0.2 (95% CI: 0.098 to 0.42); *p* < 0.001], occupation [OR = 0.65716 (95% CI: 0.65715 to 0.65718); p < 0.001], marital status [OR = 0.54 (95% CI: 0.51 to 0.57); p < 0.001], level of education [OR = 1.52 (95% CI: 1.39 to 1.67); p < 0.001], knowledge of partner’s HIV status [OR = 2.37 (95% CI: 1.07 to 5.23); *p* = 0.03] and willingness to test together with partner [OR = 0.44 (95% CI: 0.34 to 0.58); p < 0.001]. However, the outcome of multivariate analysis only revealed a significant association between uptake of CHTC and the study arm as illustrated in Table 3. Participants who were in the intervention arm had eight times higher odds of taking up CHTC compared to those in the comparison arm [AOR = 8.01 (95% CI: 2.75 to 23.32); *p* < 0.001].

**Table 3:** Multivariate analysis on factors associated with uptake of CHTC

Outcome variable Categories	Odds Ratio	95% CI	*P* value	Adj. Odds Ratio	95% CI	*P* value
Study arm						
Control	Reference					
Intervention	0.2	(0.098–0.42)	0.000*	8.01	(2.75–23.32)	0.000*
Age (years)						
18–24	Reference					
25–34	1.24	(0.76–1.2)	0.139	0.92	(0.33–2.51)	0.866
35–44	0.72	(0.25–2.12)	0.553	0.71	(0.2–2.59)	0.607
45 and above	1	^a^	^a^	^a^	^a^	^a^
Sex of participant						
Female	Reference					
Male	1.16	(0.80–1.69)	0.426	1.25	(0.58–2.68)	0.569
Occupation of participant						
Unemployed	Reference					
Employed	0.65716	(0.65715–0.65718)	0.000*	0.55	(0.22–1.35)	0.192
Marital status						
Never married	Reference					
Married	0.54	(0.51–0.57)	0.000*>	1.16	(0.46–2.89)	0.757
Widowed/separated/divorced	1.11	(0.65–1.87)	0.703	3.25	(0.59–17.78)	0.174
Education						
Primary education and below	Reference					
Secondary education	0.47	(0.12–1.92)	0.294	0.47	(0.16–1.4)	0.175
Tertiary	1.52	(1.39–1.67)	0.000*	0.58	(0.18–1.84)	0.354
Knowledge of partner’s status						
No	Reference					
Yes	0.65	(0.22–1.96)	0.446	1.06	(0.41–2.77)	0.905
Not sure if status has changed	2.37	(1.07–5.23)	0.03*	1.71	(0.48–6.05)	0.408
Willingness to invite partner for a HIV test						
No	Reference					
Yes	0.74	(0.2–2.7)	0.646	0.92	(0.26–3.32)	0.901
Willingness to test together with partner						
No	Reference					
Yes	0.44	(0.34–0.58)	0.000*	1.19	(0.25–5.65)	0.831

Note: *p < 0.05. The odds ratio are adjusted for the study arms

^a^values omitted from the model

### Qualitative findings on factors associated with uptake of CHTC

The qualitative interviews with the HTC service providers and clients revealed some of the factors associated with uptake of CHTC following the CSD intervention in the study. The clients noted that the CSD counselling equipped them with information on the benefits of CHTC which they utilised to convince their partners to go for a mutual test.

*“I broke it down for him slowly then I told him… I told him the advantages we were given on being tested together and he accepted to come for CHTC… I told him it is good for us to know our status while we are together, it was quite challenging but he agreed to come”* ***(Female, participant)***

Lack of readiness to take a HIV test with partner was implicated as a factor that impeded the uptake of CHTC.

*“…the turn up if I compare to the percentages that we were expecting the index clients to come back is a little bit low because not all of them are ready to come with their partners for CHTC…”* ***(Male, service provider)***

In some cases, the unwillingness of partners to come for CHTC following invitation by their partners also deterred the uptake of CHTC.

*“I received a reminder and I told him* [partner] *that I was supposed to go back with my partner for HIV testing...but the problem is my partner refused to come”* ***(Female, participant)***

The service providers reported that some participants were unable to come for CHTC due to other commitments as illustrated below

*“…they [index clients] were still giving excuses either they were committed, they were busy….”* ***(Female, Service provider)***

It was also reported that some participants provided wrong contact information to the service providers. Given this, they were not found on phone during follow up. This could have impeded the uptake of CHTC.

## Discussion

The uptake of CHTC was higher by 21% in the intervention arm compared to the comparison arm of the study. This was slightly above the hypothesised 20% point difference between the intervention and comparison arms of the study. The findings of the multivariate analysis also revealed that participants in the intervention arm of the study had eight times higher odds of taking up CHTC compared to those in the comparison arm. Additionally, the qualitative findings revealed that the CSD intervention improved the uptake of CHTC in the intervention sites. This finding affirmed WHO’s recommendation of CSD as a key strategy to enhance mutual testing and disclosure among couples [7]. At 28%, the rate of CHTC uptake in our study was higher than the 6% and 10% increase that was recorded by Farquhar et al. and Semrua et al. respectively in studies that employed community outreach activities to enhance the uptake of CHTC in ANC settings [17, 18]. The increase was also higher than 6% and 14.3% increase in the uptake of CHTC that was recorded in studies by Wall et al. and Allen et al. respectively who utilised influential networks to enhance the uptake of CHTC. The difference observed in our study could have been due to the empowerment of index clients on the importance of CHTC, skills on partner invitation and telephone follow up that was embedded in the model. These factors also emerged as key reasons for increased uptake of CHTC from the qualitative findings of our study. The CSD intervention equipped participants with skills to invite their partners for the uptake of CHTC through behaviour rehearsal techniques. According to WHO, behaviour rehearsal techniques in HTC settings have been shown to assist clients in developing skills that they can use in disclosing their status [8]. Similarly, the study participants reported that the skills obtained on partner invitation enabled them to invite their partners for HIV testing.

The intervention also provided clients with information on the importance and benefits of mutual testing and disclosure of HIV. This may have resulted in increased awareness on CHTC among the participants. According to Ayuo et al. *2009,* CHTC awareness was found to increase the uptake of CHTC in a study conducted in Kenya on the determinants in HIV counselling and testing in couples [14]. The qualitative findings of the study demonstrated that the increased awareness on the benefits of CHTC influenced clients to initiate discussions with their partners on mutual testing and disclosure of HIV. Additionally, Gumbo (2015) found that prior discussions on HIV led to an increase in the uptake of CHTC as was illustrated by our study [10]. Similarly, Muhindo et al.*,* 2015 also found that discussing CHCT with partner and awareness of CHCT benefits were predictors of testing among couples [26].

The CSD intervention also comprised of phone follow-up to remind participants to invite their partners for CHTC. The telephone reminders to clients to invite their partners for CHTC in our study may have prompted them to reach out to their partners. This also emerged as a factor for increased uptake of CHTC as was depicted in the qualitative interviews with clients. Additionally, a study conducted by Rosenberg et al. which employed telephone follow up and physical tracing found that partner invitation plus tracing resulted in higher uptake of CHTC compared to invitation only in a study that targeted male partners for CHTC uptake [13].

Although a high percentage (above 80%) of participants expressed willingness to take a test together with their partners in both arms of the study, this did not translate to actual uptake of CHTC as was depicted by our findings. This could have been due to unavailability of partners to test together, lack of time to test together, the unwillingness of partners to come for a HIV test and the lack of readiness to take a HIV test with the partner as was depicted by the qualitative findings. This was similar to findings from the studies by Membe (2011) and Muhindo et al. (2015) who found that lack of time and unwillingness to test together with partners negatively influenced the uptake of CHTC [11]. Some participants also gave wrong contact information and could not be traced during follow-up. This may have reduced the uptake of CHTC among the study participants.

## Conclusions

The CSD model enhanced the uptake of CHTC. Factors that were positively associated with the uptake of CHTC included being in the study intervention, counselling on the benefits of CHTC, skills on partner invitation and follow-up for partner invitation. Unwillingness of partner to test for HIV, lack of availability to test together as a couple, and provision of wrong contact information by the clients were negatively associated with the uptake of CHTC. With the increased uptake of CHTC following the intervention, the CSD model can be integrated into existing HTC strategies to improve on the uptake of mutual testing and disclosure of HIV among couples. The model can also be essential in identifying individuals who are HIV positive but have not sought HIV testing services. There is, however, need for strategies to strengthen follow-up mechanisms and to enhance contact tracing in order to yield maximum results.

## Abbreviations

CHTC: Couple HIV Testing and Counselling
CHV: Community Health Volunteer
CSD: Counsellor Supported Disclosure
FSW: Female Sex Workers
HIV: Human Immunodeficiency Virus
HTC: HIV Testing and Counselling
IPV: Intimate Partner Violence
KAIS: Kenya AIDS Indicator Survey
MSM: Men who have sex with men
PMTCT: Prevention of mother to child transmission
PrEP: Pre-exposure prophylaxis
PWID: People who inject drugs
STI: Sexually Transmitted Infections
VCT: Voluntary Counselling and Testing
WHO: World Health Organization
